# Traditional Chinese medicines in the treatment of hepatocellular cancers: a systematic review and meta-analysis

**DOI:** 10.1186/1756-9966-28-112

**Published:** 2009-08-12

**Authors:** Ping Wu, Jean Jacques Dugoua, Oghenowede Eyawo, Edward J Mills

**Affiliations:** 1 Shanghai Hospital #4, Shanghai, PR China; 2 Graduate Department of Pharmaceutical Sciences, Leslie Dan Faculty of Pharmacy, University of Toronto, Toronto, Canada; 3 Faculty of Health Sciences, Simon Fraser University, Burnaby, Canada; 4 Faculty of Health Sciences, University of Ottawa, Ottawa, Canada

## Abstract

**Background:**

Liver cancer is a common malignancy with a high mortality rate. Given the poor prognosis associated with this cancer, many patients seek additional therapies that may improve quality of life or survival. Several Traditional Chinese Medicines (TCM) have been evaluated in clinical trials, but little is known about them outside of China.

**Methods:**

We searched independently and in duplicate 8 electronic databases, including 2 Chinese language databases, until February 2009. We included any randomized clinical trials (RCT) evaluating a TCM oral preparation for the treatment of hepatocellular cancers. We abstracted data on survival, tumor response, and performance scores. We conducted a random-effects meta-analysis and applied a meta-regression analysis.

**Results:**

We included 45 RCTs (n = 3,236). All studies employed an active control group. In general, the reporting of methodological issues was poor. We analyzed data from 37 trials reporting on complete response effects score (Relative Risk [RR] of 1.26 (95 CI, 1.04–1.52, P = 0.01, I^2 ^= 0%, P = 0.99). Products containing ginseng, astragalus and mylabris had a larger treatment effect (OR 1.34, 95% CI, 1.04–1.71, P = 0.01) than the pooled broad estimate, also the case for astragalus-based treatments (OR 1.35, 95% CI, 1.001–1.80. P = 0.048). We examined survival rates and pooled 15 studies reporting on 6 month outcomes (RR 1.10, 95% CI, 1.04–1.15, P = < 0.0001, I^2 ^= 0%, P = 0.60). This effect was consistent at other prospective dates, including 12 months (22 trials, RR 1.26, 95% CI, 1.17–1.36, P = < 0.0001, I^2 ^= 7%, P = 0.36), 24 months (15 trials, 1.72, 95% CI, 1.40–2.03, P = < 0.0001, I^2 ^= 0%, P = 0.75); and, at 36 months (8 trials, RR 2.40, 95% CI, 1.65–3.49, P = < 0.0001, I^2 ^= 0%, P = 0.62).

**Limitations:**

All included trials were conducted in China where emerging evidence suggests many RCTs are not, in fact, randomized. Publication bias may exist, favouring positive reports.

**Conclusion:**

Our meta-analysis displays compelling evidence of effectiveness for hepatocellular cancers that should be evaluated in high-quality and transparent clinical trials.

## Introduction

Worldwide, liver cancer is the fifth most common malignancy in men and the eighth in women[1]. According to the World Health Organization (WHO), liver cancer is a major health problem and its incidence is increasing[2]. In the United States alone, it is estimated that there will be 22,620 new cases and 18,160 deaths related to liver cancer in 2009[3].

The major risk factor for liver cancer is the presence of cirrhosis of the liver, largely due to chronic hepatitis C virus (HCV) and hepatitis B virus (HBV) infection[4]. It is believed that the combined effects of these infections account for well over 80% of liver cancer cases worldwide[1]. Through HBV vaccines and screening of blood and blood products for HBV and HCV, primary liver cancer is the first human cancer largely amenable to prevention[1].

With respect to treatment, the plan depends on a number of factors, including the extent of the disease, growth pattern of the tumour and hepatic functional reserve of the patient[5]. In cases of localized resectable liver tumours, standard treatment is surgical resection (partial hepatectomy) in patients without liver cirrhosis and surgical resection or liver transplantation in patients with liver cirrhosis[5]. In cases of localized non-resectable liver tumours, the standard treatment of total hepatectomy with liver transplantation is considered first followed by other options, including chemoembolization, percutaneous ethanol injection, radiofrequency ablation, inclusion in clinical trials or systemic chemotherapy (anthracyclines, cisplatin and 5-FU)[5]. Systematic chemotherapy, however, is reported to have a 10% response rate and no survival benefit[5]. In cases of advanced liver tumours, there is no established standard of care[5].

Given the poor prognosis associated with some liver cancers and limited treatment options outside of surgery, patients may seek alternative treatments, including traditional Chinese medicine (TCM) products, alone or in combination with standard of care. The purpose of this study is to systematically review and meta-analyze data from randomized clinical trials (RCTs) for evidence on the efficacy of TCM products in the treatment of liver cancer.

## Methods

### Search strategy, trials selection, and data retrieval

To be eligible for inclusion in our systematic review, studies had to have enrolled adult patients (>18 years) with liver cancer. The patients had to be randomly allocated to an active TCM formulation treatment or a control group with either placebo or no treatment. In addition, any co-intervention had to be the same in both groups except for the TCM formulation. We excluded studies that reported only laboratory values rather than clinical responses. We also excluded direct comparisons of TCM formulations.

PW and EM worked independently, in duplicate, searching the following English electronic databases: MEDLINE (1966–February 2009), AMED (1985–February 2009), Alt Health Watch (1995–February 2009), CINAHL (1982–February 2009), Nursing and Allied Health Collection: Basic (1985–February 2009), Cochrane Database of Systematic Reviews (2008). In addition, PW, and YL, fluent in Mandarin and Cantonese, searched the Chinese database CNKI (1979–February 2009) and Wan Fang (1994–February 2009) independently. No language restrictions were placed on the searches.

Three reviewers (PW, EM and JL) assessed eligibility based on the full text papers and conducted data extraction, independently, using a standard pre-piloted form. Disagreements were resolved by consensus or by a third reviewer. If the required information was not available in the published article, we obtained additional information in correspondence with the authors. We included all evaluated outcome measures including: disease stage, Karnofsky performace (KP), the Child-Pugh score and the response evaluation criteria in solid tumors (RECIST). The response is categorized as complete response (CR), partial response (PR) outcomes, stable disease (SD), progressive disease (PD) and as CR + PR as a proportion for response rate (RR). We additionally examined survival rates by group according to 6, 12, 18, 24, 36 and 60-month survival rates, where reported.

In addition, we extracted data on trial quality, protocol, and outcomes assessed. We assessed quality through the reporting of the following criteria: sequence generation, allocation concealment, reporting of who was blinded, adequate descriptions of patient withdrawal, language of publication, and exposure to chemotherapy. We also noted the language in which the paper was written and the setting the studies were conducted. These criteria were not used for weighting covariates in the meta-analysis; instead, these were considered *a priori *explanations for study heterogeneity.

### Statistical analysis

We applied the Relative Risk and 95% Confidence Intervals as our primary effect measure in this analysis. For analysis examining response and survival, favourable results for the TCM intervention are in the direction greater than 1. In circumstances of zero outcome events in either arm of a trial, we used the Haldane method and added 1 to each arm, as suggested by Sheehe[6]. We first pooled studies on all interventions versus all controls using the DerSimonian-Laird random effects method[7]. This method recognizes and anchors studies as a sample of all potential studies, and incorporates an additional between-study component to the estimate of variability. We calculated the I^2 ^statistic for each analysis as a measure of the proportion of the overall variation that is attributable to between-study heterogeneity[8]. Forest plots are displayed for the primary analysis, showing individual study effect measures with 95% CIs and the overall DerSimmonian-Laird pooled estimate. We conducted a meta-regression analysis using the unrestricted maximum likelihood method to determine if the *a priori *covariates of TCM formulation yielded differing effects. We examined publication bias visually and through the Begg-Mazumdar, Egger, and Horbold-Egger tests. We calculated the optimal information size (OIS) required to determine adequate power across trials. We used Stats Direct and Comprehensive Meta-Analysis (Version 2) for all statistical procedures. All p-values are 2-sided and a p-value < 0.05 was considered significant. PW and EM conducted the analysis.

## Results

Our extensive searching yielded 130 titles and/or abstracts, of which 54 were found likely to be relevant. Nine of the full text articles reviewed were excluded for one of two reasons: 1) either the study was not randomized; 2) TCM was the control intervention 3)study was duplicated.

**Figure 1:**
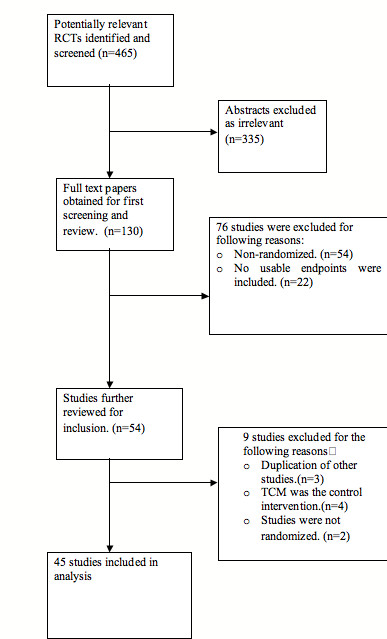
**Flow diagram of included studies**.

In total, 45 publications [9-53] containing independent data fit the criteria for inclusion. Figure 1 details the literature retrieval process used during our searches and the rationales for exclusion leading to the final selection. Among the final 45 studies, 44 [9-14,16-53]were published in Chinese languages and 1 [15]was published in English. All the studies were conducted in China.

### Characteristics of included studies

The 45 RCTs included 3,236 patients, 1,682 in the treatment groups and 1,554 in the control groups (See Additional file 1 and 2). Most trials were small and the median intervention group size was 32 (interquartile range [IQR] 30–42) and control group size is 31 (IQR 30–38). The majority of trials (24)[9,12,13,15-18,25,27,29,30,32,34-36,40,46,47,49-54] included patients with stage II or more advanced cancers. Additional file 1 displays the study characteristics and formulations along with the TCM philosophy for the preparation. All studies employed transcatheter arterial chemoembolization (TACE) as adjunct therapy. No placebo was used as the control group in any study.

### TCM Interventions

The TCM interventions identified in this study were principally combinations of different herbal medicines or animal/insect extracts (Additional file 1). A brief outline on the oncologic and immunologic pharmacology of the most commonly used ingredients is presented below.

#### Astragalus

Astragalus appears to have a number of immunomodulatory properties [55-57]. Astragalus appears to have anti-tumour activity where its potentiates LAK cell activity *in vitro *when used in combination with IL-2[58]. Astragalus appears to restore *in vitro *T-cell function, which is suppressed in cancer patients[59].

#### Panax ginseng

*Panax ginseng *and its chemical constituents were found to have inhibitory effects on putative carcinogenesis mechanisms, e.g., cell proliferation and apoptosis, immunosurveillance and angiogenesis[60]. Ginsenosides from *Panax ginseng *have been shown to inhibit tumor cell invasion and to suppress sister chromatid exchanges in human lymphocytes[61].

#### Toad skin secretions (bufotoxin)

The toad skin secretion bufalin was found to induce apoptosis in human-leukemia cells by altering expression of apoptotic genes c-myc and bcl-2[62]. Other toad skin secretions like 3-formyloxyresibufogenin, 19-oxobufalin, 19-oxodesacetylcinobufagin, 6-hydroxycinobufagin and 1-hydroxybufalin were found to exert inhibitory effects on KB, HL-60 and MH-60 cancer cell lines[63].

#### Beetle extracts (Mylabris)

An extract from *Mylabris phaleratais*, the dried body of the Chinese blister beetle, was shown to have anti-cancer activity via inducing cancer cell apoptosis and was associated with little toxicity[64].

#### Atractylodes

Atractylodes appears to have anticancer activity by inducing apoptosis and cytotoxic effects against leukemia and other cancer cell lines[65].

#### Bupleurum

Saikosaponins from *Bupleurum falcatum *were shown to exhibit potent anti-cell adhesive activity on solid tumour cells and to have strong hemolytic action[66].

#### Curcuma

*Curcuma longa *may have immunostimulatory activity[67].

### Meta-analysis

#### Complete Response

**Figure 2:**
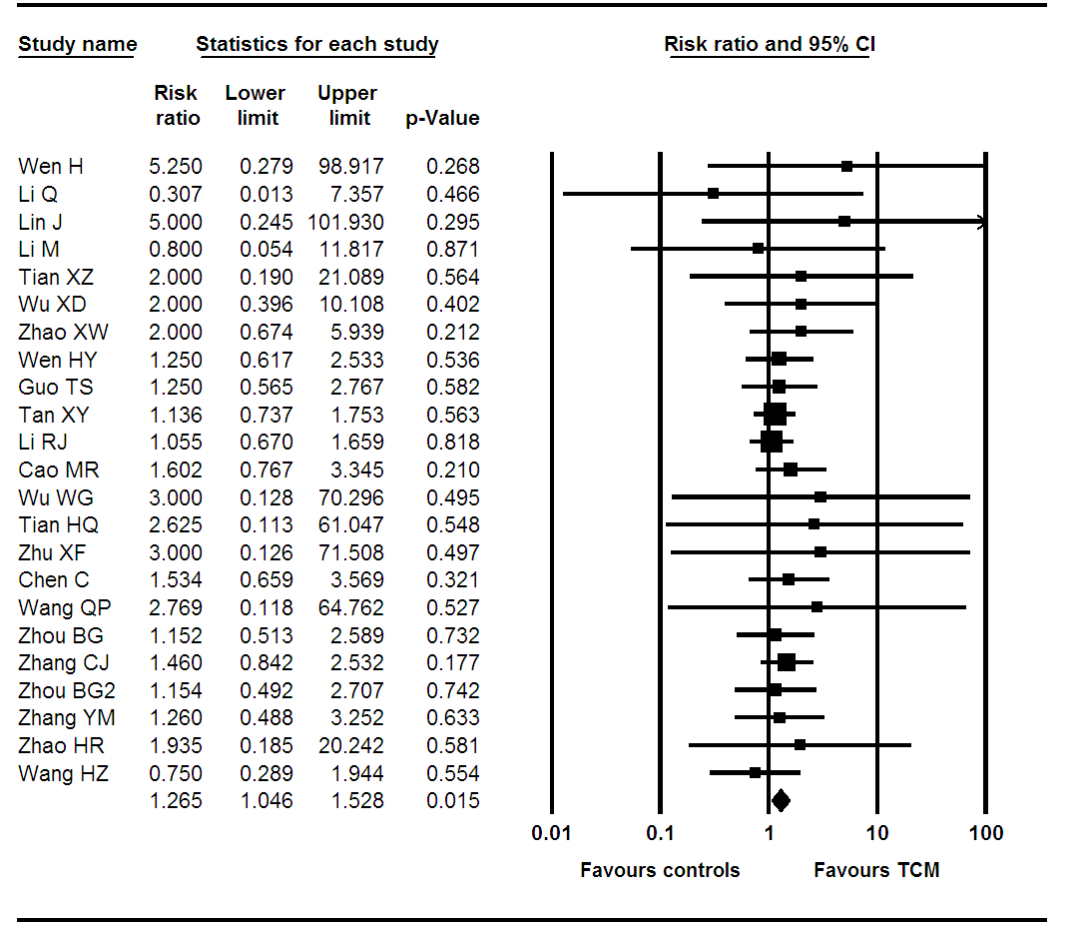
**Forest-plot of complete response**.

We analyzed data from 37 trials[10,12,13,15-18,20,21,23,25-30,32,33,35,36,38-41,44-54,68,69] reporting on RECIST CR score. Our pooled analysis indicates an RR of 1.26 (95 CI, 1.04–1.52, P = 0.01, I^2 ^= 0%, P = 0.99). See figure 2. Applying meta-regression, we found that products containing ginseng, astragalus and mylabris had a larger treatment effect (OR 1.34, 95% CI, 1.04–1.71, P = 0.01) than the pooled broad estimate and that any product containing astragalus also had this effect (OR 1.35, 95% CI, 1.001–1.80. P = 0.048).

#### Partial response

**Figure 3:**
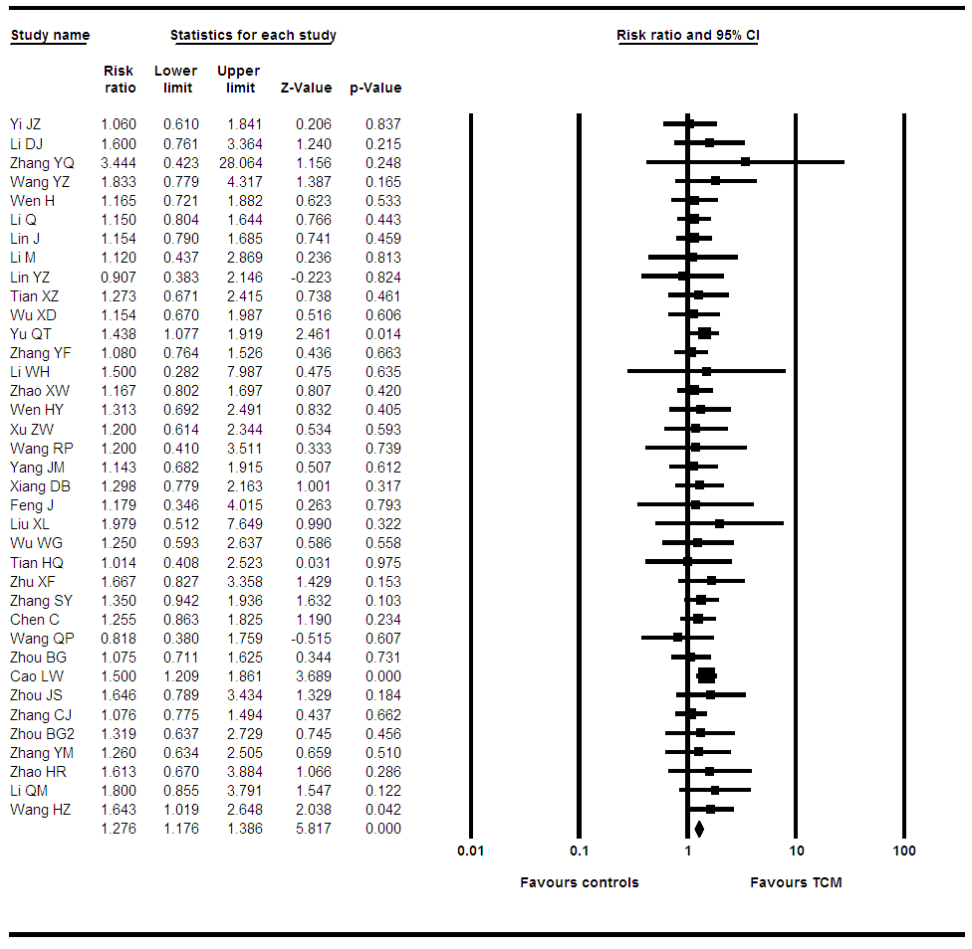
**Forest plot of partial response**.

We pooled data from 37 trials [10,12,13,15-18,20,21,23,25-30,32,33,35,36,38-41,44-54,68,69] reporting on PR between groups. The pooled RR is 1.27 (95% CI, 1.17–1.38, P = < 0.0001, I^2 ^= 0%, P = 0.99, See Figure 3). When we examined if differential effects existed across specific formulations, we found that studies using bufotoxin demonstrated increased effects (OR 1.25, 95% CI, 1.15–1.37, P = < 0.0001), as did studies using ginseng, astragalus and mylabris (OR 1.27, 95% CI, 1.16–1.39, P = < 0.0001) and any product using astragalus (OR 1.27, 1.13–1.42, P = < 0.0001).

#### Stable disease

**Figure 4:**
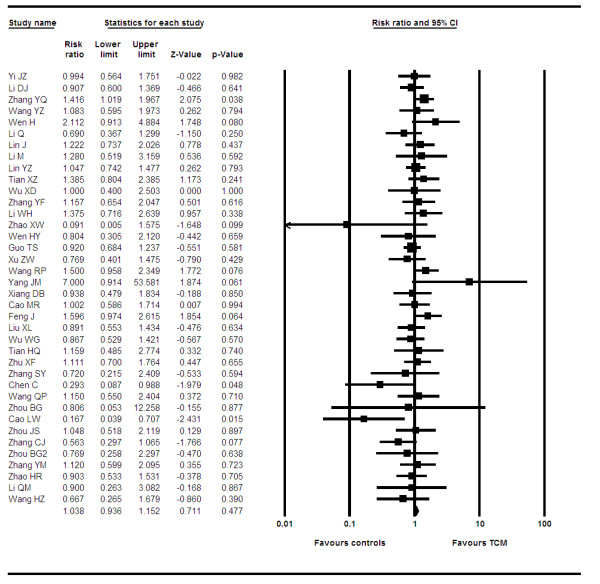
**Forest plot of stabilized disease**.

We pooled data from 37 trials[10-13,15-18,20,21,23,25-30,32,33,35,36,38-40,44-54,68,69] reporting on stable disease between groups at study conclusion. The pooled RR is 1.03 (95% CI, 0.93–1.15, P = 0.47, I^2 ^= 10%, P = 0.29, see figure 4). When we examined the effects of different preparations we did not show an effect with bufotoxin (OR 1.04, 95% CI, 0.95–1.15, P = 0.35), with ginseng, astragalus and mylabris (OR 1.04, 95% CI, 0.95–1.14, P = 0.40) or any product using astragalus (OR 1.02, 10.92–1.13, P = 0.63).

#### Progressive disease

**Figure 5:**
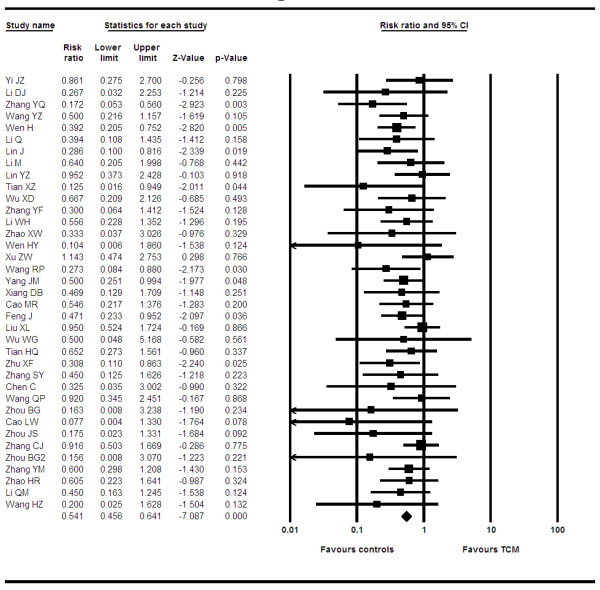
**Forest plot of progressive disease**.

We pooled data from 37 trials[11-13,15-18,20,21,23,25-30,32,33,35,36,38-40,44-54,68-70] reporting on progressive disease among patients. We found an inflated progressive disease rate in the control groups (RR 0.54, 95% CI, 0.45–0.64, P = < 0.0001, I^2 ^= 0%, P = 0.66, see figure 5). Studies utilizing bufotoxin had a decreased risk (OR 0.54, 95% CI, 0.46 to -0.65, P = < 0.0001), this was also the case with studies using ginseng, astragalus and mylabris (0.54, 95% CI, -0.46 to -0.66, P = < 0.0001) and with studies using any form of astragalus (OR 0.57, 95% CI, 0.46 to -0.70, P = < 0.0001).

### Survival rates

**Figure 6:**
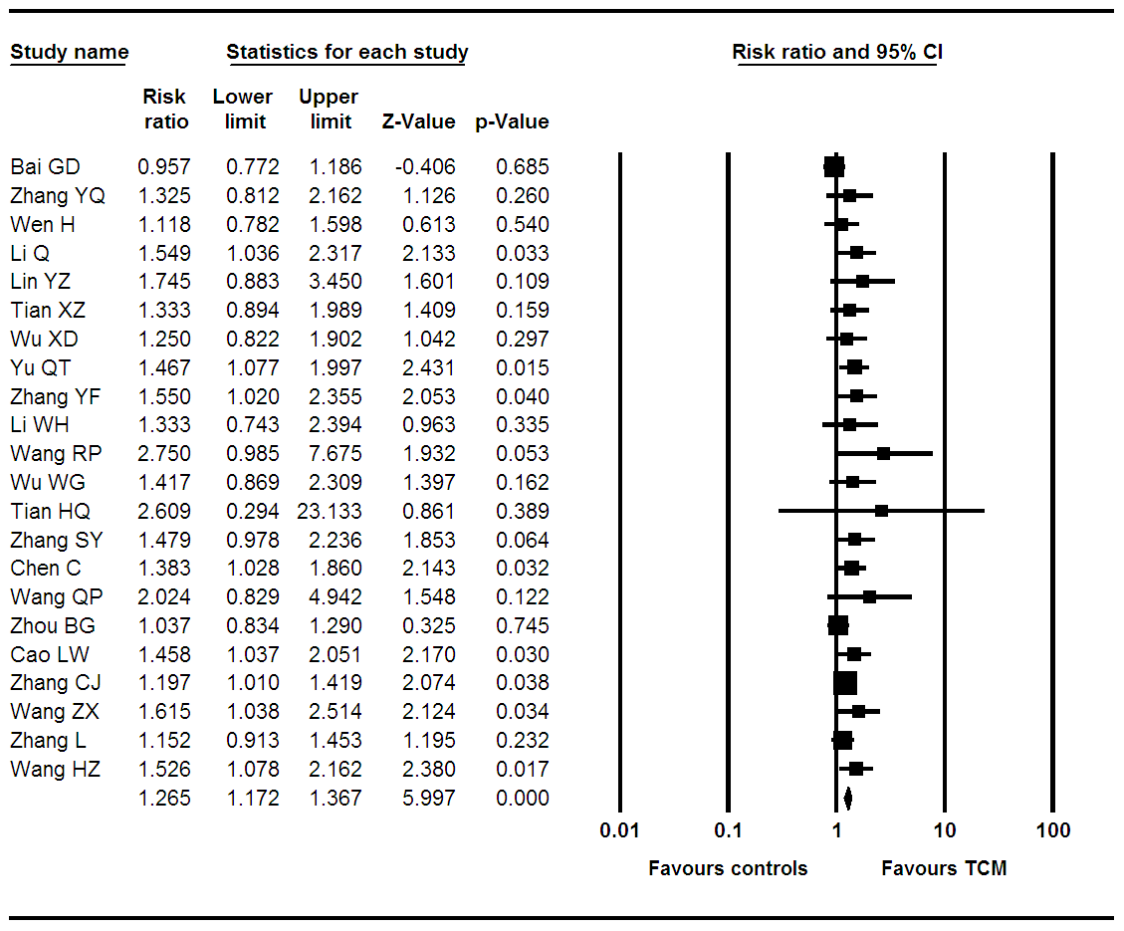
**Forest plot of 12-months survival**.

We examined survival rates and pooled 15 studies[12,17,25,26,28,29,33,36,42,44,46,50,54,69,70] reporting on 6 month outcomes (RR 1.10, 95% CI, 1.04–1.15, P = < 0.0001, I^2 ^= 0%, P = 0.60). This effect was consistent at other prospective dates, including 12 months (22 trials[9,12,17,20,25-29,31,33,35,36,41,42,44,46,47,50,54,69,70], RR 1.26, 95% CI, 1.17–1.36, P = < 0.0001, I^2 ^= 7%, P = 0.36, See figure 6); 18 months (4 trials[9,26,28,52], RR 1.71, 95% CI, 1.002–2.91, P = 0.049, I^2 ^= 70%, P = 0.009); 24 months (15 trials[17,20,26-28,31,33,36,41,42,46,52,54,69,70], 1.72, 95% CI, 1.40–2.03, P = < 0.0001, I^2 ^= 0%, P = 0.75); and, at 36 months (8 trials[27,31,33-35,42,47,69], RR 2.40, 95% CI, 1.65–3.49, P = < 0.0001, I^2 ^= 0%, P = 0.62). We applied meta-regression on the 12 month survival and found increased effect with bufotoxin (OR 1.22, 95% CI, 1.13–1.32, P = < 0.0001) and with products containing ginseng, astragalus and mylabris (OR 1.24, 95% CI, 1.16–1.33, P = < 0.0001) and astragalus alone (OR 1.28, 95% CI, 1.15–1.40, P = < 0.0001).

### Symptom improvement

Several studies reported on improvement of symptoms. In particular, 6 studies[13,15,23,29,44,68] reported on abdominal pain improvements favouring TCM approaches (RR 1.50, 95% CI, 1.09–2.07, P = 0.013, I^2^44%, P = 0.11). Abdominal distension did not improve among TCM recipients in 5 reported trials^8,18,24,39,50 ^(RR 1.26, 95% CI, 0.96–1.64, P = 0.09, I^2 ^= 4%, P = 0.38). Fatigue significantly improved in 4 reported trials^8,18,24,39^, (RR 1.54, 95% CI, 1.17–2.01, P = 0.001, I^2 ^= 0%, P = 0.87), and appetite improved in 4 reported trials^8,18,24,39^, (RR 1.53, 95% CI, 1.14–2.05, P = 0.004, I^2 ^= 0%, P = 0.45).

### Optimal Information Size (OIS)

Almost all trials included in our analysis were small. We applied OIS based on the event rate in the intervention and control arms for the PR outcome. We found an event rate of 0.42 in the intervention arms and an event rate of 0.33 in the control arms. When applying 80% power and a two-tailed 5% alpha, we identify that we require at least 906 participants in our meta-analysis.

### Publication bias

We assessed publication bias visually with a funnel plot and applied several statistical tests to determine the likelihood of publication bias. We found no vidence when applying the Begg-Mazumdar test (P = 0.14), Egger's test (P = 0.80) or Horbold-Egger's test (P = 0.89). We also imputed the number of studies that were likely missing, but the resulting number was unconcerning (n = 2) and was unlikely to change the effect estimate.

## Discussion

We found consistent effects of traditional Chinese medicines when combined with TACE versus TACE alone. The majority of studies included in our analysis were small or of moderate size and none can provide definitive answers on treatment options, although compelling results related to bufotoxin, astragalus and products containing ginseng, astragalus and mylabris warrant further examination. Our study also highlights the utility that searching in non-English languages may have on identifying potentially useful new interventions for common diseases. While our study finds compelling results, there is also reason for caution, given the poor reporting of clinical trials in China. Only independently conducted research from high-quality research teams will strengthen the inference of effectiveness.

Strengths of our study include our extensive searches of literature in both English and in Chinese languages, and using Chinese language databases for our search. Two of us (PW, JL) understand and read Mandarin and Cantonese, along with English, thus allowing searches across several languages. We applied a broad criteria for pooling studies. We included any TCM formulation and then conducted a meta-regression analysis to determine if specific preparation yielded differing effects over the broad group, and in several cases did.

Limitations of our study include the underlying concern about the quality of the included studies. As we highlight, the majority of studies were small, with typically 30 participants per arm. Meta-analysis aims to overcome issues of power through pooling, thus increasing sample size and power. We applied an OIS on the overall event rate of partial response and found that a pooled sample size of 1,108 provided sufficient evidence of an effect. This did not apply to specific formulations. We further assessed issues of methodological rigour as two major concerns with Chinese-based clinical trials. Firstly, is that only positive trials are published in Chinese medical journals, and second, is that some trials reported as randomized are, in fact, not randomized. A recent evaluation by Wu et al. found that many studies labelled as RCTs with Chinese journals were, in fact, not randomized[71] In our own experience, we recognize many Chinese clinical trialists have not been exposed to appropriate clinical epidemiology training. We examined publication bias through both visual inspection of the funnel plot on the primary outcome (PR) and through statistical tests, but were unable to identify publication bias. However, funnel plots cannot rule out publication bias and we remain cautious that many negative trials likely exist.

From a clinical standpoint, the results of this study are very encouraging but should be implemented with caution. The average clinician will be reassured that TCM interventions, both herbal-based and animal/insect-based, were safely combined with chemotherapy. The average clinician, however, likely will not scrutinize the results of this study using evidence-based principles and may implement our findings into practice due to the overwhelming positive response in our meta-analysis. Given this tendency, the results from this study should be carefully disseminated to the medical community with the caveat that although promising, our findings need to be confirmed via a RCT conducted in a Western academic setting.

Our study may prove useful for a number of reasons. Firstly, there is reason to further examine the evidence of several of the interventions included in our analysis. Other investigators have examined the role of herbal medicines and TCM interventions for hepatocellular cancers, lung cancers and hepatitis and found compelling evidence in humans [72-75] However, perhaps a far more important finding from our analysis and approach is the role that searching for clinical trials in non-English languages may play in drug discovery. Important first line drugs, such as artemisin-based therapies for malaria, have been discovered through searching existing trials in non-English languages. [76]

There have now been two studies prior to ours that examined the role of TCM interventions on survival and clinical outcomes in patients also receiving TACE. [72,75] The first study, by Shu et al[72], published in 2005, included 26 RCTs of interventions including 2079 patients. Similar to ours, they found improved survival at 12 months [RR 1.55, 95% CI, 1.39–1.72], at 24 months [RR 2.15, 95% CI, 1.75–2.64], and at 36 months [RR, 2.76, 95% CI, 1.95–3.91]. Tumor response was also significantly increased [RR 1.39, 95% CI, 1.24–1.56]. A more recent study, published in 2009 by Cho and Chen, [75] included 30 studies including 2428 patients. As with ours, they found increased survval at 12 months [OR 1.92, 95% CI, 1.43–2.57], at 24 months [OR 3.55, 95% CI, 2.36–5.36], and at 36 months [OR 5.12, 95% CI, 2.76–9.52]. The inflated effect sizes found in the study by Cho and Chen may be related to their choice of effect size of OR rather than the more conservative RR((([77] Given that all three reviews found compelling evidence of a role for TCM in hepatocellular cancers, it seems appropriate that further evaluations, in a non-Chinese setting, occur in order to determine if we have a possible new opportunity for drug development.

Our study builds on the findings of others about the heterogeneous quality of randomized trials from China. In our own experience in China, we have doubts that many methodological features attributed to randomized trials, were in fact conducted. A previous analysis, by Vickers et al, found that most trials conducted in China were reported as positive,[78] a finding our analysis also supports^8^. While several explanations for this phenomenon exist, a likely explanation is the slow uptake of evidence-based medicine and clinical trials methodology in academic research centres[79] With the opening of the Chinese Cochrane Centre, we hope that clinical epidemiology will receive considerably more attention[80]

In conclusion, our study provides important inferences about new potential therapeutic options for hepatocellular cancers. While these finds are compelling, there is a need for confirmation of these studies in well-conducted RCTs conducted in Western settings. Until such time, potentially useful interventions cannot be wholly recommended based on evidence alone.

## Competing interests

The authors declare that they have no competing interests.

## Authors' contributions

PW, JJD, EM conceived the study. PW, JJD, EM, OE participated in protocol design. PW, JJD, EM, OE ran the searches and abstracted data. EM performed the analysis. PW, JJD, EM, OE wrote and approved the manuscript.

## Supplementary Material

Additional file 1 **Characteristics of included studies**. Table describing characteristics of study populations and interventions. Click here for file

Additional file 2 **Ingredients and TCM philosophy for each study**. Table describing individual ingredients and TCM philosophy for the use of the ingredients. Click here for file
