# Supplementary material for: Traditional Chinese medicines in the treatment of hepatocellular cancers: a systematic review and meta-analysis
Source: J Exp Clin Cancer Res. 2009 Aug 12;28(1):112. doi: 10.1186/1756-9966-28-112 (PMC3225807; doi:10.1186/1756-9966-28-112)
Supplement: Additional file 1 — Characteristics of included studies. Table describing characteristics of study populations and interventions. [file 1756-9966-28-112-S1.doc]

| **author** | **year** | **n** | | **clinical stage** | **Child score** | **KPScore** | **common Treatment** | **TCM in Tr.Group** | **Chemo Intervention times** | **TCM duration (days)** |
| --- | --- | --- | --- | --- | --- | --- | --- | --- | --- | --- |
|  |  | **Tr.** | **Cont'** |  |  |  |  |  |  |  |
| Lin YZ ^22^ | 2005 | 52 | 33 | II, III | A, B | >=60 | TACE | Shen Tao Ruan Gan Bolus | 2 | 60 |
| Tian XZ ^26^ | 2006 | 36 | 36 | NA | NA | 〉70 | TACE | Ai Yi Shu injection | 2-3 | 60-90 |
| Wu XD ^36^ | 2003 | 30 | 30 | II, III | A, B | >=50 | TACE | Hu Gan Ruan Jian Fang | 1 | 48 |
| Yu QT ^41^ | 2004 | 64 | 64 | NA | NA | NA | TACE | Chinese toad bufotoxin injection | 2--3 | 60-90 |
| Zhang YF ^46^ | 2000 | 50 | 45 | II, III | NA | NA | TACE | Chinese herbal compound | 2--3 | 〉60 |
| Li WH ^20^ | 2006 | 19 | 19 | NA | NA | NA | TACE | Chinese toad bufotoxin injection | 2 | 60 |
| Zhao XW ^49^ | 2005 | 30 | 30 | II | NA | NA | TACE | Shen Qi capsule | 2 | 56 |
| Wen HY ^32^ | 2006 | 32 | 30 | II, III | NA | NA | TACE | Chinese herbal compound | 2.3 | >90 |
| Guo TS ^14^ | 2005 | 33 | 33 | NA | NA | NA | TACE | Jew Ear Parasitized Granula | 1 | 30 |
| Xu ZW ^38^ | 2000 | 30 | 30 | NA | NA | NA | TACE | [modified six nobles decoction decoction](javascript:showjdsw('jd_t','j_')) | 1 | 14 |
| Wang RP ^29^ | 2002 | 30 | 30 | II, III | NA | >60 | TACE | Gan Ji grain | 2 | 90-120 |
| Yang JM ^39^ | 2006 | 31 | 31 | NA | NA | NA | TACE | Ai Di injection | 2.5 | 38 |
| Tan XY ^24^ | 2005 | 33 | 30 | NA | NA | >60 | TACE | Ai Di injection | 2 | 40 |
| Li RJ ^19^ | 2005 | 32 | 30 | NA | NA | >=60 | TACE | Ai Di injection | 2 | 42 |
| Xiang DB ^55^ | 2006 | 32 | 30 | NA | NA | NA | TACE | De Li Shen injection | 2 | 40 |
| Cao MR ^11^ | 2003 | 29 | 38 | NA | A, B, C | NA | TACE | Ai Di injection | 1.8 | 18 |
| Feng J ^13^ | 2002 | 35 | 33 | II, III | NA | NA | TACE | Chinese herbal compound | 2--3 | 90-180 |
| Liu XL ^23^ | 2002 | 32 | 38 | NA | NA | NA | TACE | Yan Shu injection | 1 | 15 |
| Wu WG ^35^ | 2001 | 25 | 25 | I， II， III | A, B | 〉=60 | TACE | [pingxiao capsule](http://dict.cnki.net/dict_result.aspx?searchword=平消胶囊&tjType=sentence&style=&t=pingxiao+capsule) | 2 | 90 |
| Tian HQ ^25^ | 2001 | 23 | 20 | II, III | NA | 60-80 | TACE | Chinese herbal compound | 2 | 60 |
| Zhu XF ^53^ | 2006 | 40 | 40 | I， II， III， IV | A, B, C | >60 | TACE | Kang lai Te injection | 2 | 42 |
| Zhang SY ^44^ | 1996 | 30 | 27 | NA | NA | NA | TACE | AC-III injection | 2 | >30 |
| Chen C ^12^ | 2001 | 42 | 41 | II, III | NA | NA | TACE | Pei Ben Gu Yuan anti-cancer capsule | 3 | 90 |
| Wu JX ^34^ | 1999 | 13 | 12 | II | NA | NA | TACE | Yi Gan Jian |  |  |
| Wang QP ^28^ | 2006 | 25 | 23 | NA | NA | NA | TACE | Ai Di injection | 3 | 120 |
| Zhou BG ^50^ | 1999 | 31 | 25 | II, III | NA | NA | TACE | [pingxiao capsule](http://dict.cnki.net/dict_result.aspx?searchword=平消胶囊&tjType=sentence&style=&t=pingxiao+capsule) | 3 | >90 |
| Cao LW ^57^ | 2005 | 50 | 50 | NA | A | NA | TACE | Gan Fu Kang Capsule | 3--4 | 60-80 |
| Zhou JS ^52^ | 2006 | 21 | 22 | II, III | A，B， C | 50-70 | TACE | Chinese toad bufotoxin injection | 4 | 120 |
| Zhang CJ ^56^ | 2005 | 116 | 108 | NA | A，B， C | NA | TACE | Jin Long capsule | 4 | 1095 |
| Zhou BG ^51^ | 2002 | 26 | 20 | II, III | NA | NA | TACE | Pingxiao capsule | 3 | 90 |
| Wang ZX ^31^ | 2001 | 63 | 44 | NA | NA | NA | TACE | Fuzhenhuaji detoxification pill | 3 | 63 |
| Zhang YM ^45^ | 2005 | 50 | 42 | NA | NA | NA | TACE | Shanxian Granula | 3 | 90 |
| Zhao HR ^48^ | 2004 | 31 | 30 | NA | NA | NA | TACE | Jew Ear Parasitized Granula | 3 | 60 |
| Li QM ^18^ | 2003 | 20 | 18 | II, III | NA | >=60 | TACE | Qining injection | 3 | 21 |
| Zhang L ^42^ | 2005 | 60 | 50 | NA | A，B， C | >=60 | TACE | Qingganjiedusanjie decoction | 4 | 180-300 |
| Wang HZ ^27^ | 1998 | 36 | 36 | II, III | A，B， C | NA | TACE | Chinese herbal compound | 3--7 | 60-150 |
| Yi JZ ^40^ | 2008 | 36 | 31 | II, III | NA | >60 | TACE | Kang Ai injection | 3 | 45 |
| Li DJ ^15^ | 2009 | 30 | 32 | II, III | NA | >=60 | TACE | Kanglaite capsule | 3 | >60 |
| Bai GD ^9^ | 2008 | 26 | 26 | II, III | A, B | >70 | TACE | Chinese herbal compound | 1.3 | 30 |
| Zhang YQ ^47^ | 2008 | 45 | 31 | II, III, IV | NA | NA | TACE | Xiao Yao San | 2.5 | 7d before TACE |
| Wang YZ ^30^ | 2008 | 30 | 30 | I, II, III | NA | NA | TACE | Qing Gan Hua Yu oral liquid | 1 | 30 |
| Wen H ^33^ | 2008 | 55 | 41 | NA | A, B | NA | TACE | Chinese herbal compound | >2 | >30 |
| Li Q ^17^ | 2008 | 50 | 46 | I, II, III | A, B | >=60 | TACE | Chinese toad bufotoxin injection | 3.2 | 4 |
| Lin J ^21^ | 2008 | 58 | 58 | NA | NA | >=50 | TACE | Fuzhen detoxification decoction | 1 | 60 |
| Li M ^16^ | 2007 | 20 | 16 | II, III | NA | 70(mean) | TACE | Chinese herbal compound | 2 | >60 |
