# Supplementary material for: Traditional Chinese medicines in the treatment of hepatocellular cancers: a systematic review and meta-analysis
Source: J Exp Clin Cancer Res. 2009 Aug 12;28(1):112. doi: 10.1186/1756-9966-28-112 (PMC3225807; doi:10.1186/1756-9966-28-112)
Supplement: Additional file 2 — Ingredients and TCM philosophy for each study. Table describing individual ingredients and TCM philosophy for the use of the ingredients. [file 1756-9966-28-112-S2.doc]

| **Author** | **TCM in Tr.Group** | **Ingredients** | **TCM philosophy** |
| --- | --- | --- | --- |
|
| Lin YZ ^22^ | Shen Tao Ruan Gan Bolus | Artemisia capillaris,Hedyotis diffusa Willd,Scutellaria barbata,Rhizoma Curcuma,Peach Kernel, Angelica Sinensis,salvia,white ginseng,Poria Cocos, Tortoise Plastron | supplement Qi, strengthening spleen,replenishign blood, blood-activiating and stasis-dissolving, clearing heat and removing dampness and detoxification |
| Tian XZ ^26^ | Ai Yi Shu injection | Disodium Cantharidinate Injectio | breaking stagnant and eliminating blood stasis; attacking toxin and eliminating and resolving stagnation and masses |
| Wu XD ^36^ | Hu Gan Ruan Jian Fang | radix codonopsis, Astragalus , poria cocos, Hedyotis diffusa Willd， bear grass， Centipede，Atractylodes，Curcuma，Bupleurum，CARAPAX TRIONYCIS | supplement Qi, strengthening spleen,replenishign blood,eliminate and resolve stagnation and masses, nourishing kidney,supporting the normal and raising of the origin |
| Yu QT ^41^ | Chinese toad bufotoxin injection | Chinese toad bufotoxin injection | detoxification, detumescence and analgesia, eliminate and resolve stagnation and masses |
| Zhang YF ^46^ | Chinese herbal compound | Bupleurum， Astragalus， red radix paeoniae alba, Atractylodes, Magnolia, Szechwan Chinaberry Fruit, Membrane of Chicken Gizzard, wolfberry fruit, | relieving the depressed liver,invigorating spleen and stomach, nourishing liver and kidney,eliminate and resolve stagnation and masses |
| Li WH ^20^ | Chinese toad bufotoxin injection | Chinese toad bufotoxin injection | detoxification, detumescence and analgesia, eliminate and resolve stagnation and masses |
| Zhao XW ^49^ | Shen Qi capsule | Angelica Sinensis, lanceolata,Schisandra Chinensis, Artemisia capillaris,Astragalus,medofenoxate Extra,milk thistle | supplement Qi,nourishing Yin and liver,invigorating spleen and kidney, |
| Wen HY ^32^ | Chinese herbal compound | American ginseng, CARAPAX TRIONYCIS, Zedoary, rhizome sparganii, tortoise plastron, White Peony Root, Astragalus | supplement Qi,nourishing Yin and liver,invigorating spleen and kidney,eliminate and resolve stagnation and masses, relieving pain |
| Guo TS ^14^ | Jew Ear Parasitized Granula | Jew Ear Parasitized | strengthening vitals,activating blood and eliminating broomsymptom |
| Xu ZW ^38^ | [modified six nobles decoction](javascript:showjdsw('jd_t','j_')) | ginseng,Atractylodes, Poria Cocos, Glycyrrhiza, Tangerine Peel,Pinellia ternate,Perilla,Radix auckladiae, Citrus Aurantium,areca peel | supplement Qi and invigorating spleen; eliminating dampness to reducephlegm |
| Wang RP ^29^ | Gan Ji grain | Astragalus, CARAPAX TRIONYCIS, lanceolata, Coix seed, White Peony Root, Atractylodes, Zedoary, barbed skullcap | supplement Qi and eliminate and resolve stagnation and masses |
| Yang JM ^39^ | Ai Di injection | ginseng, Astragalus,Mylabris,medofenoxate | clearing heat,detoxification ,eliminate and resolve stagnation and masses |
| Tan XY ^24^ | Ai Di injection | ginseng, Astragalus,Mylabris,medofenoxate | clearing heat,detoxification ,eliminate and resolve stagnation and masses |
| Li RJ ^19^ | Ai Di injection | ginseng, Astragalus,Mylabris,medofenoxate | clearing heat,detoxification ,eliminate and resolve stagnation and masses |
| Xiang DB ^55^ | De Li Shen injection | red ginseng,Astragalus,Mylabris,arenobufagin | supplement Qi and strengthening vitals, eliminate and resolve stagnation and masses |
| Cao MR ^11^ | Ai Di injection | ginseng, Astragalus,Mylabris,medofenoxate | clearing heat, detoxification ,eliminate and resolve stagnation and masses |
| Feng J ^13^ | Chinese herbal compound | ginseng,Atractylodes, Poria Cocos, Glycyrrhiza, Tangerine Peel,Pinellia ternate,Perilla,Radix auckladiae, Citrus Aurantium,areca peel | detoxification, detumescence and analgesia, eliminate and resolve stagnation and masses |
| Liu XL ^23^ | Yan Shu injection | Sophora flavescens,white Poria Cocos | clearing heat and detoxification, removing dampness; |
| Wu WG ^35^ | [pingxiao capsule](http://dict.cnki.net/dict_result.aspx?searchword=平消胶囊&tjType=sentence&style=&t=pingxiao+capsule) | Curcuma Longa,Alumen,Potassium nitrate, nux vomica,Agrimony,etc | clearing heat and detoxification; eliminate and resolve stagnation and masses,Strengthening Healthy Qi |
| Tian HQ ^25^ | Chinese herbal compound | Lanceolata, Astragalus, Coix seed, Bupleurum, Common Yam Rhizome, Reynoutria, Akebia Stem | invigorating spleen and liver; clearing heat removing dampness; Strengthening Healthy Qi; |
| Zhu XF ^53^ | Kang lai Te injection | Coix seed oil injection | strengthening spleen,invigorating the lung, clearing heat and removing dampness; |
| Zhang SY ^44^ | AC-III injection | Ginseng, Arsenolite, calomelas, muskmelo pedicel | Strengthening Healthy Qi;removing blood stasis,toxicity and excessive pathogenic factors |
| Chen C ^12^ | Pei Ben Gu Yuan anti-cancer capsule | Ginseng, Corydalis, Astragalus, Salvia, balsam pear, Mythic Fungus, Hedyotis diffusa Willd，Angelica, Algae, acanthopanax senticosus, Scutellaria barbata | strengthening vitals; eliminate and resolve stagnation and masses, relieving pain; promoting digestion and anchoring mind; replenishing qi and blood; clearing heat and detoxification; promoting urination and dehumidification; |
| Wu JX ^34^ | Yi Gan Jian | Radix Adenophorea , radixcodonopsis,radix ophiopogonis, angelica, radix rehmanniae, polyporus, solanum nigrum, prunella vulgaris, Spreading Hedvotis Herb etc. | clearing heat and nourishing Yin, strengthening spleen and removing dampness,replenishing qi and blood, cooling blood to stop bleeding. |
| Wang QP ^28^ | Ai Di injection | ginseng, Astragalus,Mylabris,medofenoxate | clearing heat,detoxification ,eliminate and resolve stagnation and masses |
| Zhou BG ^50^ | [pingxiao capsule](http://dict.cnki.net/dict_result.aspx?searchword=平消胶囊&tjType=sentence&style=&t=pingxiao+capsule) | Curcuma Longa,Alumen,Potassium nitrate, nux vomica,Agrimony,etc | clearing heat,detoxification ,eliminate and resolve stagnation and masses,Strengthening Healthy Qi |
| Cao GW ^57^ | Gan Fu Kang Capsule | Ginseng, Astragalus, Scorpion, atractylodes , cyperus rotundus, placenta hominis, Scutellaria barbata | strengthening vitals; replenishing qi and blood; clearing heat, detoxification |
| Zhou JS ^52^ | Chinese toad bufotoxin injection | Chinese toad bufotoxin injection | detoxification, detumescence and analgesia, eliminate and resolve stagnation and masses |
| Zhang CJ ^56^ | Jin Long capsule | fresh bungarus parvus, Gekko | breaking stagnant and eliminating blood stasis;resolveing depression and dredging meridian |
| Zhou BG ^51^ | pingxiao capsule | Curcuma Longa,Alumen,Potassium nitrate, nux vomica,Agrimony,etc | clearing heat,detoxification ,eliminate and resolve stagnation and masses,Strengthening Healthy Qi |
| Wang ZX ^31^ | Fuzhenhuaji detoxification pill | dark plum, ginseng, atractylodes, CARAPAX TRIONYCIS, sophora flavescens, Poria Cocos, Coix seed, Dogwood Fruit, Scutellaria barbata | strengthening body resistance, eliminate and resolve stagnation and detoxification |
| Zhang YM ^45^ | Shanxian Granula | Hawthorn,Agrimony,American Ginseng, Zedoary,etc | replenishign blood,eliminate and resolve stagnation and masses, strengthening spleen and supplement Qi |
| Zhao HR ^48^ | Jew Ear Parasitized Granula | Jew Ear Parasitized Granula | strengthening vitals,activating blood and eliminating broomsymptom |
| Li QM ^18^ | Qining injection | Disodium Cantharidinate Injection | breaking stagnant and eliminating blood stasis; attacking toxin and eliminating and resolving stagnation and masses |
| Zhang L ^42^ | Qingganjiedusanjie decoction | radix codonopsis, bupleurum, Scutellaria barbata, Astragalus, red peony root, paris rhizome, Hedyotis diffusa Willd, centipede, curcuma longa | clearing liver, heat and detoxification, eliminate and resolve stagnation and masses |
| Wang HZ ^27^ | Chinese herbal compound | Astragalus, poria cocos, wolfberry fruit, raw glycyrrhiza | strengthening liver ,kidney and supplementing Qi; clearing heat and detoxification; promoting urination and dehumidification; |
|  | | | |
| Yi JZ ^40^ | Kang Ai injection | Astragalus, ginseng, KushensuMarine | supporting the healthy energy and supplementing the vital energy |
| Li DJ ^15^ | Kanglaite capsule | semen coisis | supplement Qi and nourish Yin, eliminate and resolve stagnation and masses |
| Bai GD ^9^ | Chinese herbal compound | red ginseng, Astragalus,Hedyotis diffusa Willd,poria cocos,Atractylodes,white peony root,Angelica Sinensis, rhizomaligusticichuanxiong, prepared radix rehmanniae, Laminaria japonica,seaweed,barbed skullcap | strengthening spleen and Replenishing kidney, clearing away heat and detoxification,soft the hard lumps and dissipate phlegm |
| Zhang YQ ^47^ | Xiao Yao San | bupleurum,white peony root,atractylodes, angelica sinensis, poria cocos,baked licorice,mint, achyranthes root | soothing liver and strengthening spleen, strengthening body resistance |
| Wang YZ ^30^ | Qing Gan Hua Yu oral liquid | Not mentioned in detail | strengthening spleen and supplementing Qi, eliminate and resolve stagnation and masses,clearing away heat and detoxification |
| Wen H ^33^ | Chinese herbal compound | white peony root,Astragalus,parched pangolin scales, zedoary turmeric, toosandan fructus,paris rhizome | supplement Qi and nourish Yin, eliminate and detoxification |
| Li Q ^17^ | Chinese toad bufotoxin injection | Chinese toad bufotoxin injection | detoxification, detumescence and analgesia, eliminate and resolve stagnation and masses |
| Lin J ^21^ | Fuzhenhuaji detoxification decoction | Atractylodes,Hedyotis diffusa Willd, lanceolata,poria cocos, barbed skullcap,red peony root,Red Sage Root, herba artemisiae scopariae | strengthening body resistance and spleen, supplementing Qi, eliminate and resolve stagnation and masses |
| Li M ^16^ | Chinese herbal compound | white peony root,Atractylodes,lanceolata, citrus aurtantium, cortex moutan,peach kernel, Indian Bread | strengthening spleen and supplementing Qi,blood-activiating and stasis-dissolving |
